# Characterization of antimicrobial substance from *Lactobacillus salivarius* KL-D4 and its application as biopreservative for creamy filling

**DOI:** 10.1186/s40064-016-2693-4

**Published:** 2016-07-12

**Authors:** Phatthanaphong Therdtatha, Chanabhorn Tandumrongpong, Komkhae Pilasombut, Hiromi Matsusaki, Suttipun Keawsompong, Sunee Nitisinprasert

**Affiliations:** Specialized Research Unit, Probiotics and Prebiotics for Health, Department of Biotechnology, Faculty of Agro-Industry, Kasetsart University, Bangkok, 10900 Thailand; Center for Advanced Studies for Agriculture and Food (CASAF), Kasetsart University Institute for Advanced Studies (NRU-KU), Kasetsart University, Bangkok, 10900 Thailand; Division of Animal Production Technology, Faculty of Agricultural Technology, King Mongkut’s Institute of Technology Ladkrabang, Bangkok, 10520 Thailand; Department of Food and Health Sciences, Faculty of Environmental and Symbiotic Sciences, Prefectural University of Kumamoto, Kumamoto, 862-8502 Japan

**Keywords:** *Lactobacillus salivarius*, Salivaricin, Bakery products, Creamy filling, Biopreservative

## Abstract

*Lactobacillus salivarius* KL-D4 isolated from duck intestine produced bacteriocin which was stable at high temperature and a wide pH range of 3–10. Its cell free supernatant at pH 5.5 exhibited wide inhibitory spectrum against both G+ and G− bacteria. The highest bacteriocin production was obtained in MRS broth supplemented with 0.5 % (w/v) CaCO_3_ at 6 h by gentle shaking. PCR walking using specific primers at the conserved region of class-II bacteriocin resulted in 4 known genes of *kld*1, *kld*2, *kld*3 and *kld*4 with 100 % similarity to genes encoding for salivaricin α, β, induction peptide and histidine protein kinase of *Lb. salivarius* GJ-24 which did not previously report for bacteriocin characterization, while showing 94, 93, 59 and 62 % to other salivaricin gene cluster, respectively. The high activities of 25,600 AU/ml indicated a strong induction peptide expressed by *kld3* which has low similarity to previous inducer reported. Based on operon analysis, only *kld*1, *kld*3 and *kld*4 could be expressed and subsequently elucidated that only salivaricin α like bacteriocin was produced and secreted out of the cells. Using protein purification, only a single peptide band obtained showed that this strain produced one bacteriocin which could be salivaricin α namely salivaricin KLD showing about 4.3 kDa on SDS-PAGE. Partial purification by 20 % ammonium sulfate precipitation of the product was tested on the artificial contamination of creamy filling by *Bacillus cereus*, *Enterococcus faecalis*, *Pseudomonas stutzeri*, *Staphylococcu*s sp. and *Stenotrophomonas* sp. resulting the growth inhibitory efficiency of 4.45–66.9, 11.5–100, 100, 0–28.1 and 5–100 % respectively. Therefore, salivaricin KLD can be a tentative biopreservative for food industry in the future.

## Background

Bakery products usually contain basic elements of milk, butter, starch, eggs and sugar that provide the nutrient sources for the good growth of microbial contaminants (Pyler 1988). However, these food products are generally considered to be microbiologically safe due to the high temperature of up to 170 °C used in the baking process. Usually, baked products do not pose a health risk unless they have cream or custard filling containing egg or a dairy product (Stewart et al. 2003). This filling is the most likely source of a serious food safety hazard due to post-baking microbial contamination. Another risk may arise from climatic conditions facilitating microbial growth, especially in a tropical country like Thailand. Several antimicrobial substances, such as acetic acid, propionic acid, sorbic acid and their salts have been used as food preservatives to extend a product’s shelf life (Smith et al. 2004). However, their inhibition efficiencies at the neutral pH of bakery products with creamy filling have been reported as low which corresponded to previous studies proposed that chemical preservatives are only effective in bakery products at pH < 6.5 (Guynot et al. 2005; Rosenquist and Hansen 1998). Schmidt et al. (1969) reported that both potassium sorbate and sodium benzoate at a concentration of 1000 ppm could inhibit the growth of *Staphylococcus aureus* in synthetic cream pies at 22 and 37 °C and at pH 4.5–5.0 but not at a higher pH of 5.2. In addition, it was found that calcium propionate shows no inhibition against rope-producing spores containing *B. subtilis*, *B. licheniformis* and *B. pumilus* in white bread (Thompson et al. 1998).

Recently, biopreservatives produced by lactic acid bacteria (LAB) have been of interest due to consumer concerns regarding chemical additives in food products. Bacteriocin, one of effective biopreservative compounds, is a ribosomally synthesized peptide that has been investigated and widely applied for food preservative in the bakery industry (Chen and Hoover 2003; Galvez et al. 2007). Enterocin AS-48, which is produced by *E. faecalis* A-48-32, is able to partially reduce the populations of spoilage and potential endospore-forming bacilli in wheat dough stored at 22 °C at a concentration of 14 AU/g (Viedma et al. 2011). *Lactobacillus salivarius,* one alternative from bacteriocin producing strains is potential to be the biopreservative for bakery products. This species is usually reported as a probiotic candidate frequently isolated from human, porcine, and avian gastrointestinal tracts (Dobson et al. 2012). It often produces bacteriocin belonging to class II, which typically exhibits antibacterial activity against spoilage bacteria, as well as food-borne pathogens (Messaoudi et al. 2013). Salivaricin ABP-118, which is produced by *Lb. salivarius* UCC118, demonstrates a broad-spectrum of antibacterial activity, including the Gram-positive bacteria of *Listeria*, *Staphylococcus*, *Enterococcus* and *Bacillus* species (Dunne et al. 1999). Salivaricin FK22, which is produced by *Lb. salivarius* K7, displays inhibitory activity against Gram-positive bacteria (Pilasombut et al. 2006). It is also reported that salivaricin CRL1328, which is produced by *Lb. salivarius* CRL1328, shows bacteriocin activity against potential bacteria causing food-poisoning such as *E. faecalis* and *E. faecium* (Ocaña et al. 1999).

In our laboratory, *Lb. salivarius* KL-D4 isolated from duck intestine was preliminarily investigated and found that it was able to produce antimicrobial substance (AMS) against potential foodborne pathogen. Therefore, given such potential efficiency, this study aimed to characterize the AMS produced by the strain KL-D4 and further investigate its application as a biopreservative in creamy filling.

## Methods

### Bacterial strains and culture conditions

*Lactobacillus salivarius* KL-D4 was used as an AMS producer strain and cultivated in MRS broth (Difco, USA) at 37 °C. All target bacterial strains and their growth conditions used in this study were listed in Table 1. All strains were maintained at −80 °C in appropriate media containing 20 % (v/v) glycerol (Univar, Australia). *Escherichia coli* DH5α, which was used as a host for cloning, was grown in Luria–Bertani broth (Sambrook et al. 2001) supplemented with 100 μg/ml of amplicillin (Amresco, USA) at 37 °C with vigorous agitation for 16–18 h.

**Table 1:** List of target bacterial strains and their cultivation conditions

Indicator strain	Media	Temperature (°C)
Lactic acid bacteria		
*Lactococcus lactis* subsp. *cremoris* TISTR1344	MRS	30
*Lactobacillus plantarum* ATCC14917	MRS	30
*Lactobacillus sakei* subsp. *sakei* JCM1157^T^	MRS	30
*Lactobacillus sakei* TISTR890	MRS	30
*Leuconostoc mesenteroides* subsp. *mesenteroides* JCM6124	MRS	30
*Leuconostoc mesenteroides* subsp. *mesenteroides* TISTR942	MRS	30
*Enterococcus fecalis* KUB-E5	NB	37
*Enterococcus fecalis* KUB-E7	NB	37
Other Gram-positive bacteria		
*Bacillus cereus* JCM2152	NB	37
*Bacillus coagulans* JCM2257	TSBYE	37
*Bacillus coagulans* TISTR1447	TSBYE	37
*Brochotrix campeatris* NBRC11547	TSBYE	25
*Listeria innocua* ATCC33090	TSBYE	37
*Staphylococcus epidermidis* KUB-E6	NB	37
*Staphylococcus hominis* KUB-E8	NB	37
*Streptococcus* sp. TISTR1030	NB	30
Gram-negative bacteria		
*Aeromonas hydrophila* TISTR1321	NB	30
*Escherichia coli* O157:H7	NB	37
*Pseudomonas fluorescens* JCM5963	TSBYE	25
*Pseudomonas fluorescens* TISTR358	TSBYE	25
*Pseudomonas stutzeri* KUB-E1	NB	37
*Salmonella enterica serova Enteritidis* DMST17368	NB	37
*Stenotrophomonas* sp. KUB-E2	NB	37

*ATCC* American Type Culture Collection, Rockville, Md; *DMST* Department of Medical Science Thailand, Thailand; *JCM* Japanese Culture of Microorganism, Wako, Japan; *NBRC* National Institute of Technology and Evaluation (NITE) Biological Resource Center; *TISTR* Thailand Institute of Scientific and Technological Research; *KUB* Department of Biotechnology, Kasetsart University, Thailand

### Determination of enzyme sensitivity

The sensitivity of the bacteriocin to proteolytic enzymes and other enzymes was performed using a reaction containing 1 ml of cell free supernatant (CFS) and 1 mg/ml of each enzyme for 2 h under the following suitable conditions: trypsin and α-chymotrypsin (Sigma-Aldrich, USA) at 25 °C and pH 8.5; α-amylase (Sigma-Aldrich, USA) at 25 °C and pH 7.0; proteinase K (Sigma-Aldrich, USA) at 37 °C and pH 8.0; actinase E (Kaken Pharmaceutical, Japan) and lipase (Sigma-Aldrich, USA) at 37 °C and pH 7.0; pepsin at 37 °C and pH 3.0 (Nacalai Tesque, Japan). After incubation, all reaction mixtures were heated at 100 °C for 5 min and the residual inhibitory activity was determined against *Lb. sakei* subsp. *sakei* JCM 1157^T^ using the spot-on-lawn method.

### Determination of growth and bacteriocin production

Bacteriocin productivity and growth determination were adapted from Flynn et al. (2002) and Yamamoto et al. (2003). One percent innoculant of KL-D4 strain was grown in 100 ml of MRS broth under four different conditions: static, static supplemented with 0.5 % (w/v) of CaCO_3_ (Scharlau, European Union), shaking at 120 rpm and shaking at 120 rpm supplemented with 0.5 % (w/v) of CaCO_3_. All broth cultures were then incubated at 37 °C for 24 h to determine their effects on cell growth and bacteriocin production. Each sample was withdrawn from its broth culture every 3 h to determine cell concentration, pH, and inhibitory activity. The viable cells were monitored in two replications by a standard plate count technique (Wehr and Frank 2004). The specific growth rate (μ) at the exponential phase was calculated equation below:

$$\upmu = \frac{{(\ln x_{t2} {-}\ln x_{t1} )}}{{\left( {t_{2} - t_{1} } \right)}}$$

where x_t1_ and x_t2_ are the viable cell concentration measured within the exponential phase of growth at times t_1_ and t_2_, respectively.

For further determination of inhibitory activity, the CFS was prepared by heating at 70 °C for 30 min and tested against *Lb. Sakei* subsp. *sakei* JCM1157^T^ by the spot-on-lawn method.

### Determination of pH and heat stability

Samples of the CFS of 16 h culture solution were adjusted to pH values of 3.0, 4.0, 5.0, 5.5, 6.0, 7.0, 8.0, 9.0 and 10.0 and incubated at 37 °C for 2 h. All samples were then adjusted to pH 5.5 and determined for their residual activities. To determine heat stability, CFS samples were adjusted to pH 5.5 and heated at 100 °C for 5 and 30 min and at 121 °C for 15 min. The remaining bacteriocin activities were determined against *Lb. sakei* subsp. *sakei* JCM 1157^T^ by the spot-on-lawn method.

### Determination of inhibitory activity

An inhibitory activity was determined using the spot-on-lawn method according to the modified method of Ennahar et al. (1999). The 1.0 % (w/v) soft agar medium, containing the target strain of approximately 10^7^ CFU/ml was overlaid on the same medium containing 1.5 % (w/v) agar. CFS was adjusted to pH 5.5 with 5 N NaOH solution (Merck, Germany) and heated at 70 °C for 30 min. Ten microliters of the sample was spotted onto the overlaid surface and the plate was incubated at an appropriate temperature for each target strain for 18 h. The activity was defined as the reciprocal of the last serial dilution giving a zone of inhibition and expressed as activity units (AU) per milliliter.

### Identification of bacteriocin by polymerase chain reaction (PCR)

Identification of genes related to the production and regulation of bacteriocin from *Lb. salivarius* KL-D4 strain was performed using the PCR walking technique detailed below. All of the primers used in the experiment were shown in Table 2.

**Table 2:** List of primers and nucleotide sequences

Primer set	Sequence 5′–3′	Purpose
SalAα-f1	5′-ATGAAGGAATTTACAGTATTGACAGAATGT-3′	To amplify *kld*1
SalAα-r1	5′-TTATAAACAAGTAAGCGCTCCGCCTACCAT-3′	
D4-f1	5′-GCTGGTATTGTAGGCGGAGCAAACTTAGGA-3′	To amplify *kld*2,3,4
Oligo105	5′-CYTCDATNGCRTTRTC-3′*	
D4-f1	5′-GCTGGTATTGTAGGCGGAGCAAACTTAGGA-3′	To amplify *kld*5
D4-r1	5′-CTACACAATTAGGATAACGTTTCCCACCGT-3	
D4-f2	5′-GATTAAGACATTCAAATCAAGATATTTGTA-3′	To confirm *kld* locus
D4-r2	5′-ATGTAACGACTATAAATACAATATTTTGTT-3′	
T7 promoter	5′-TAATACGACTCACTATAGGG-3′	Colony PCR for pT7Blue-T vector
U-19mer	5′-GTTTTCCCAGTCACGACGT-3′	
M13RV	5′-CAGGAAACAGCTATGAC-3′	Colony PCR for pMD20-T vector
M13M4	5′-GTTTTCCCAGTCACGAC-3′	

* D = A, G, T; R = A, G; Y = C, T

#### Polymerase chain reaction

Total DNA of *Lb. salivarius* KL-D4 was extracted following Sato et al. (2000) and used as a template for the PCR amplification. Firstly, PCR was performed using the forward and reverse primers SalAα-f1 and SalAα-r1, respectively, based on the nucleotide sequence of salivacin K21 (Matsusaki et al. 2006). About 50–200 ng of DNA template was amplified with 0.5 μM of each primer in 50 μl of premix solution (Takara Bio Inc, Japan), containing 1.25 units of Ex Taq HS DNA polymerase, 1× Ex Taq buffer (Mg^2+^ plus), and 0.2 mM of dNTPs. The PCR conditions were: denaturation at 94 °C for 3 min, followed by 30 cycles including denaturation at 94 °C for 1 min, annealing at 45 °C for 1 min and extension at 72 °C for 2 min. The second PCR followed was performed using an internal sense primer D4-f1 located at the internal sequence of the first PCR product and Oligo105 related to the consensus sequence of histidine protein kinase (Losteinkit et al. 2001). The reaction of the PCR mixtures (50 μl) consisted of about 50–200 ng of DNA template, 0.5 μM of D4-f1 primer, 1.5 μM of Oligo105 primer, 1.25 U of Ex Taq HS DNA polymerase, 1× Ex Taq buffer (Mg^2+^ plus) and 0.2 mM of dNTPs. The PCR conditions were: denaturation at 94 °C for 3 min, followed by 30 cycles included denaturation at 94 °C for 1 min, annealing at 53 °C for 1 min and extension at 72 °C for 3 min. PCR product obtained was analysed by agarose gel electrophoresis and further submitted for cloning and nucleotide sequence analysis.

#### Inverse PCR

An inverse PCR technique was performed using the DNA template of restriction enzyme digested chromosomal DNA and primers of D4-f1 and the internal antisense primer D4-r1 located on the internal sequence of the first PCR product. The genomic DNA was randomly cut by restriction enzymes, including *Apa*I, *Sal*I, *Ban*III, *Hin*dIII, *Ec*oRI, *Xba*I, *Xho*I, *Sac*I and *Sac*II (Takara Bio Inc, Japan). Each enzymatic treatment was performed in 400 μl of premix solution, containing 50 ng of genomic DNA, 10X buffer of each enzyme and 1 unit of the enzyme. All treatments were incubated at 37 °C for 24 h. Subsequently, the digested DNA was purified using a phenol–chloroform treatment and precipitated using cold ethanol. Self ligation of the digested DNA obtained was performed in 5 μl of Ligation high Ver.2 (Toyobo, Japan) at 16 °C for 2 h. The ligated DNA solution was purified using ethanol precipitation, dried using a Mini Vacuum-Centrifugal Evaporator (Tomy, Japan) and dissolved in 100 μl of TE buffer. To perform the inverse PCR reaction, 10 ng of each ligation product was amplified with 10 μM of each primer in 50 μl of premix solution (Takara Bio Inc, Japan) containing, 10 μM of D4-f1 primer, 10 μM of D4-r1 primer, 1.25 units of PrimeSTAR HS DNA polymerase (TaKaRa, Japan), 5× PrimeSTAR buffer (Mg^2+^ plus) and 2.5 mM of dNTPs. The amplification program was 94 °C for 3 min, 30 cycles of 98 °C for 10 s, 55 °C for 5 s, 72 °C for 10 min with a final extension at 72 °C for 20 min. PCR product obtained was analysed by agarose gel electrophoresis and further submitted for cloning and nucleotide sequence analysis.

#### Analysis of gene cluster from *Lb. salivarius* KL-D4

The gene cluster was performed using the primer D4-f2 and the antisense primer D4-R2 located on the upstream and downstream region of the cluster gene, respectively. The reaction of the PCR mixtures (50 μl) consisted of about 50–200 ng of genomic DNA used as a template, 10 μM of each primer, 1.25 units of PrimeSTAR HS DNA Polymerase, 5X PrimeSTAR buffer (Mg^2+^ plus) and 2.5 mM of dNTPs. The PCR conditions were: denaturation at 94 °C for 3 min, followed by 30 cycles included denaturation at 98 °C for 10 s, annealing at 55 °C for 15 s and extension at 72 °C for 1.30 min. PCR product obtained was analysed by agarose gel electrophoresis and further submitted for cloning and nucleotide sequence analysis.

#### Cloning and DNA sequencing

All PCR products obtained were purified using a QIAEX II kit (Qiagen, USA). The amplified PCR products performed by the set of SalAα-f1, SalAα-r1 and D4-f1, Oligo105 were cloned into pT7Blue-T vector (Novagen, USA) while the rest were cloned into pMD20-T vector (Takara, Japan). All PCR products were ligated using 5 μl of Ligation high Ver.2 at 16 °C for 2 h. Ligation products were transformed into *E. coli* DH5α according to the transformation method of Sambrook et al. (2001). The positive clones were screened using blue-white colony selection on agar plates and the recombinant plasmids were examined for the presence of the genes related to bacteriocin production using the colony PCR technique as follows. Each positive clone was suspended in 10 μl of 2X EmeraldAmp PCR Mastermix (Takara Bio Inc., Japan) and 2 μl of each primer. The PCR conditions were: denaturation at 94 °C for 5 min, 35 cycles included denaturation at 94 °C for 10 s, annealing at 55 °C for 30 s and extension at 72 °C for 2 min. The recombinant plasmid of the positive clone was extracted using a FlexiPrep™ kit (Amersham Biosciences, USA) and sequenced by the modified method of Sanger and Coulson (1975) using a LI-COR automated DNA sequencer model 4200 (Li-COR Biosciences, USA).

#### Data analysis

Database searches were performed using blastx in the BLAST program of the National Center for Biotechnology Information (NCBI; http://www.ncbi.nlm.nih.gov/BLAST). Open reading frame analysis was performed using the ORF finder program (http://www.ncbi.nlm.nih.gov/gorf/gorf.html). Peptide property predictions were performed using a peptide property calculator program (Innovagen, http://www.pepcalc.com). Nucleotide sequences alignment was performed using the GENETYX-MAC software (GENETYX^®^, Japan). Promoter prediction was performed using the Neural Network Promoter Prediction software (http://www.fruitfly.org/seq_tools/promoter.html). Protein sequences alignment was performed using the BioEdit Sequence Alignment Editor program V.7.0.0 (http://www.mbio.ncsu.edu/BioEdit/bioedit.html).

### Bacteriocin purification

#### Purification method

According to the modified method of Flynn et al. (2002), the bacteriocin was purified by three steps of purification, consisting of an ammonium sulfate precipitation, cation exchange chromatography and reverse-phase HPLC. All fractions collected were determined for bacteriocin activity using *Lb. sakei* subsp. *sakei* JCM 1157^T^ as an indicator strain.

#### Ammonium sulfate precipitation

One liter of CFS from 16 h culture grown in MRS and 0.5 % (w/v) of CaCO_3_ at 37 °C under shaking conditions at 120 rpm was heated at 70 °C for 30 min and subsequently cooled down to 4 °C. The bacteriocin precipitation was achieved using 20 % (w/v) saturated ammonium sulfate (Univa, Australia) and stirred at 4 °C for 18 h. The bacteriocin pellets were collected by centrifugation at 4400×*g* for 20 min and dissolved in 1 l of 20 mM citrate buffer pH 5.5 to dilute salt concentration.

#### Cation exchange chromatography

Cation exchange chromatography was performed using a fast protein liquid chromatography (FPLC) system (ÄKTA™ explorer, model 900, Amersham Bioscience, USA) with the Unicorn V.5.01 software. The UV detection used in this step was carried out at 220 nm. One liter of AMS solution from ammonium sulfate precipitation was applied into 20 ml of the strong cation exchange resin (SP Sepharose Fast Flow, Amersham, Sweden) packed in a XK16 column (Amersham Bioscience, USA) at a flow rate of 2 ml/min and washed with 3× column volume of 20 mM citrate buffer pH 5.5 at the flow rate of 1 ml/min. One-step elution of 20 ml buffer containing 0.8 M NaCl was used to obtain the bacteriocin solution.

#### Reverse-phase HPLC

This process was performed using the Breeze 2 System (Walter, USA), including a binary pump: model 1525 (Walter, USA), a 6 port injector: model 7725 (Rheodyn, USA), a UV/visible detector: model 2489 (Walter, USA) and a computer with the Breeze software installed. The bacteriocin separation was achieved using a Resource RPC 3 ml column (Amersham Bioscience, Sweden) at the flow rate of 1 ml/min. The injection sample volume was 5 ml. Two mobile phase systems were used and consisted of solvent A (0.1 % (v/v) trifluoroacetic acid (TFA) in deionized water) and solvent B (acetonitrile containing 0.1 % TFA). Five milliliters of bioactive fractions obtained from cation exchange chromatography was subjected to the Resource RPC column equilibrated with 3X column volume of solvent A. Elution was then carried out with a linear gradient of 100 %A/0 %B to 0 %A/100 %B for 30 min. The active fractions were subjected to RP-HPLC a second time to obtain the pure bacteriocin. The residual acetonitrile was removed by using a speed vacuum concentrator (Uivapo100H, Uniequip, Germany) before testing the bacteriocin activity and applying it for SDS-PAGE.

#### Determination of protein concentration

Protein concentrations were determined using a Bio-Rad protein Assay kit (Bio-Rad, USA), which was based on the Bradford method (Kruger 2002).

#### Tris–Tricine SDS-PAGE

An active fraction was subjected to 15 % (w/v) polyacrylamide-gel electrophoresis as described by Bollage and Edelstein (1991) using a Tris–Tricine buffer system performed according to the manufacturer’s instruction of the Mi-Protean^®^ Tetra system (Bio-Rad, USA) at 100 V for 45 min. Fermentas Spectra™ Multicolor Low Range Protein Ladder (Thermo Scientific, USA) was used to estimate the molecular weight of the pure bacteriocin. The gel was stained using a Pierce^®^ Silver stain kit (Thermo Scientific, USA).

#### Zymogram analysis

A zymogram of bacteriocin was performed as described by Losteinkit et al. (2001). After electrophoresis, the gel was divided into two parts. The first part was stained with the silver stain kit to check the bacteriocin purity. The second part was used to determine the activity against *Lb. sakei* subsp. *sakei* JCM1157^T^ by washing three times with sterilized water for 10 min and subsequently placing on MRS agar containing the target strain to obtain a clear zone.

### Preparation of bacteriocin-treated bacterial contaminating Filling

To prepare partially purified salivaricin KLD (PP-KLD), 1 L of CFS from 16 h culture grown in MRS and 0.5 % (w/v) of CaCO_3_ at 37 °C under shaking conditions at 120 rpm was heated at 70 °C for 30 min and cooled down to 4 °C. The bacteriocin was precipitated using 20 % saturated ammonium sulfate (Univa, Australia), stirring at 4 °C for 18 h and centrifuging at 4400×*g* for 20 min. Subsequently, the bacteriocin pellets obtained were dissolved in 20 ml of 20 mM citrate buffer pH 5.5 and later freeze dried (FD50 Series, Epsilon, Thailand). The freeze-drying was performed under a vacuum as follows: pre-freeze at −35 °C for 3 h, main drying at −35, 10 and 25 °C for 2, 6 and 6 h, respectively, and final drying at 35 °C for 13 h. The inhibitory activities of 800,000 AU/g against *Lb. sakei* subsp. *sakei* JCM 1157^T^ were obtained.

To prepare creamy filling, 1 g of the filling consisting of evaporated milk (0.30 g), egg (0.10 g), sucrose (0.18 g), corn flour (0.06 g) and butter (0.06 g) was mixed well at 60–70 °C for 10 min and then sterilizing by autoclaving at 121 °C for 15 min. Then, 0.3 ml of PP-KLD to obtain the final concentrations of 1, 5 and 10 % by weight of filling (w/w), 1 % (w/w) of commercial food preservative H-3 (Asama Chemical, Japan) and 2 % (w/w) of glycine (Yuki Gosei Kogyo, Japan) were added and mixed well. The concentration of commercial products used was the maximum that do not effect to the taste of products. Sterilized distilled water was added instead of AMS and used as a negative control.

To perform bacterial contamination, a single colony of each target strain was inoculated in 5 ml of nutrient broth (NB) and incubated at 37 °C for 16 h under shaking conditions at 150 rpm. Subsequently, 10 μl of each target strain was inoculated into the filling and AMS-treated filling to obtain the final cell concentration of 5 log CFU/g and then mixed well using a stomacher (Seward, England) at normal speed for 1 min. All treatments were incubated at 37 °C for 3 days. Total viable counts at 0 and 3 days were determined by the standard plate-count method (Wehr and Frank 2004). Each treatment was performed for two replications. The growth inhibitory efficiency (GIE) in the filling was evaluated according to the modified method of Wongsuttichote and Nitisinprasert (2009) using the equation:

$${\text{GIE}} = \frac{{{\text{C}}_{\text{c}} {-}{\text{C}}_{\text{t}} }}{{{\text{C}}_{\text{c}} }} \times 100$$

where C_c_ and C_t_ are log CFU of the control (without AMS) and the treatment (addition of AMS) at each condition, respectively.

### Statistical analysis

The average values from the duplicate experiments were determined using an Excel program (Microsoft Corp., USA). To determine the standard deviations and statistical significance of data, a one-way ANOVA was performed at the 95 % confidence interval (*P* < 0.05) using the SPSS statistical package version 22 (IBM Corp., USA).

## Results

### Preliminary identification of antimicrobial substance from *Lb. salivarius* KL-D4

The inhibitory activity of a KL-D4 strain was completely eliminated when the culture solution was treated with all proteolytic enzymes except amylase and lipase (Table 3). These results strongly indicated that the AMS is a proteinaceous structure corresponding to the bacteriocin definition (Klaenhammer 1993). Therefore, this AMS produced by the KL-D4 strain was a bacteriocin.

**Table 3:** Inhibitory activities against *Lactobacillus sakei* subsp*. sakei* jcm1157^T^ of cell free supernatant under various conditions

Treatment	Activity (AU/ml)
Enzymatic stability	
Control	12,800
Protenase K pH 8.0	0
Pepsin pH 3.0	0
Trypsin pH 8.5	0
α-chymotrypsin pH 8.5	0
Actinase E pH 7.0	0
α-amylase pH 7.0	12,800
Lipase pH 7.0	12,800
Heat stability	
100 °C for 5 min, pH 5.5	25,600
100 °C for 30 min, pH 5.5	25,600
121 °C for 15 min, pH 5.5	25,600
pH stability	
pH 3.0	12,800
pH 4.0	12,800
pH 5.0	12,800
pH 5.5	25,600
pH 6.0	25,600
pH 7.0	25,600
pH 8.0	12,800
pH 9.0	12,800
pH 10.0	12,800

### Effect of CaCO_3_ and agitation to cell growth and bacteriocin production

**Fig. 1:**
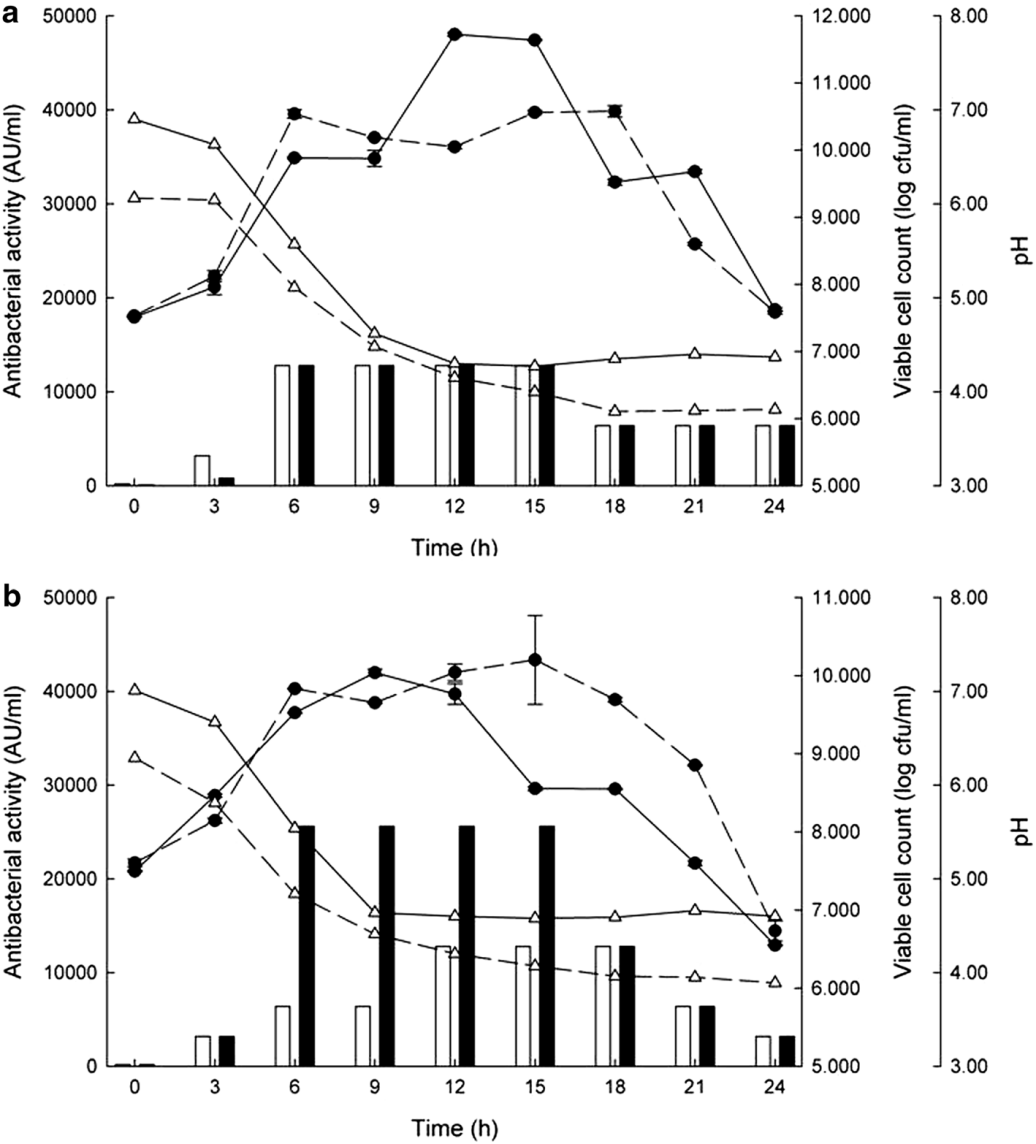
Growth and bacteriocin production by *Lb. salivarius* KL-D4 in MRS broth. Static condition (**a**); shaking condition with 120 rpm (**b**); supplementation with CaCo_3_, *solid line*; with out CaCo_3_, *dash line*; pH during fermentation, (*triangle*); viable cells determined by standard plate count method, (*black circle*); *black bars* and white ones are antibacterial activities from culture grown in MRS supplement with and without CaCO_3_, respectively

The effects of the CaCO_3_ added to neutralize the lactic acid during growth and low agitation at 120 rpm were determined under four different conditions: shaking at at low velocity of 120 rpm (SK), shaking at 120 rpm supplemented with 0.5 % (w/v) of CaCO_3_ (SK + Ca), static conditions (ST) and static conditions supplemented with 0.5 % (w/v) of CaCO_3_ (ST + Ca). The results were shown in Fig. 1a, b. The growth of all conditions were increased dramatically at log phase and approached the stationary phase since 6 h. Their viable cell concentrations decreased at 15–18 h. The ST + Ca condition showed higher viable cell than the ST condition at stationary phase. While the pH of without calcium carbonate of both conditions (3.81–3.89) decreased more than the one with calcium carbonate (4.37–4.6) consequently affecting higher survival cells of fermentation with calcium carbonate addition due to acid accumulation. However, both static conditions produced the maximum bacteriocin level in the log phase within 6 h (1.28 × 10^4^ AU/ml) and then gradually decreased in the stationary phase after 15 h (Fig. 1a). While shaking conditions with Ca produced higher bacteriocin activities of 2.56 × 10^4^ AU/ml in the middle log phase (6 h) (Fig. 1b). However, the specific growth rates of all conditions were not significantly different (*P* > 0.05) shown in Table 4. Therefore, it was clearly shown that the SK + Ca condition displayed higher bacteriocin activities than the static conditions for twofold even their growth rate were not different.

**Table 4:** Specific growth rate of bacteriocin producing *Lb. salivarius* KL-D4 grown in MRS medium with and without CaCO_3_ at 37° C under static and shaking condition

Condition	Specific growth rate (μ)
Static condition (ST)	0.09 ± 0.01
Static condition with 0.5 % (w/v) of CaCO_3_ (ST + Ca)	0.08 ± 0.01
Shaking at 120 rpm (SK)	0.06 ± 0.00
Shaking at 120 rpm with 0.5 % (w/v) of CaCO_3_ (SK + Ca)	0.04 ± 0.00

### Effect of pH and heat treatment

The results are shown in Table 3 with stability over a wide pH range of 3.0–10.0 with 100 % stability of 25,600 AU/ml in the pH range of 5.5–7.0. Moreover, the bacteriocin activities of 25,600 AU/ml at 100 °C for 5 and 30 min and by autoclaving at 121 °C for 15 min still remained at 100 %.

### Inhibitory spectrum of cell free supernatant and partially purified bacteriocin

The CFS levels adjusted to pH 5.5 were determined for inhibitory activity against 17 bacterial strains of both Gram-positive and Gram-negative bacteria (Table 5). All lactic acid bacteria (LAB) tested were inhibited. The most sensitive strain was *Lb. sakei* subsp. *sakei* JCM1157^T^. However, the bacteriocin could not inhibit the growth of *Bacillus coagulans* TISTR1447, *Brochotrix campeatris* NBRC11547, *Streptococcus* sp. TISTR1030, *Aeromonas hydrophila* TISTR1321, *Stenotrophomonas sp.* KUB-E2, *Escherichia coli* O157:H7 and *Salmonella enterica* serovar Enteritidis DMST17368.

**Table 5:** Inhibitory activities of cell free supernatant and partial purified salivaricin KLD against various target strains

Indicator strain	Inhibitory activity (AU/ml)
Lactic acid bacteria^1^	
*Lactococcus lactis* subsp. *cremoris* TISTR1344	800
*Lactobacillus plantarum* ATCC14917	200
*Lactobacillus sakei* subsp. *Sakei* JCM1157^T^	25,600
*Lactobacillus sakei* TISTR 890	100
*Leuconostoc mesenteroides* subsp. *mesenteroides* JCM6124	400
*Leuconostoc mesenteroides* subsp. *mesenteroides* TISTR942	800
Other Gram-positive bacteria^1^	
*Bacillus cereus* JCM2152	3200
*Bacillus coagulans* JCM2257	100
*Bacillus coagulans* TISTR1447	0
*Brochotrix campeatris* NBRC11547	0
*Listeria innocua* ATCC33090	6400
*Streptococcus* sp. TISTR1030	0
Gram-negative bacteria^1^	
*Aeromonas hydrophila* TISTR1321	0
*Escherichia coli* O157:H7	0
*Pseudomonas fluorescens* JCM5963	800
*Pseudomonas fluorescens* TISTR358	800
*Salmonella enterica serova Enteritidis* DMST17368	0
Bacterial contaminants from Éclair product^2^	
*Enterococcus fecalis* KUB-E5	12,800
*Enterococcus fecalis* KUB-E7	12,800
*Staphylococcus epidermidis* KUB-E6	800
*Staphylococcus hominis* KUB-E8	3200
*Pseudomonas stutzeri* KUB-E1	1600
*Stenotrophomonas* sp. KUB-E2	0

The strain KUB-E1, -E2, -E5, -E6, -E7 and -E8 isolated from éclair production process and deposited in theculture collection of the Specialized Research Unit: Probiotics and Prebiotics for Health, Department of Biotechnology, Kasetsart University, Thailand

*ATCC* American Type Culture Collection, Rockville, Md; *DMST* Department of Medical Science Thailand, Thailand; *JCM* Japanese Culture of Microorganism, Wako, Japan; *NBRC* National Institute of Technology and Evaluation (NITE) Biological Resource Center; *TISTR* Thailand Institute of Scientific and Technological Research

^a^Activities determination of CFS

^b^Activities determination of KLD

Partially purified salivaricin KLD (PP-KLD) using 20 % (w/v) saturated ammonium sulfate precipitation exhibited high activities against *Enterococcus faecalis* KUB-E5 and *E. faecalis* KUB-E7, which were isolated from the éclair production process. It was interesting that bacteriocin in the form of CFS and partially purified bacteriocin exhibited inhibitory activities of 800 and 1600 AU/ml, respectively, against *Pseudomonasfluorescens* and *P. stutzeri* which were Gram-negative bacteria as well.

### Identification of bacteriocin from *Lb. salivarius* KL-D4 by polymerase chain reaction

*Lactobacillus salivarius*, the candidate of the bacteriocin producer strain, was discovered many years ago (Messaoudi et al. 2013). Some of these species could produce a bacteriocin named salivacin or salivaricin (Barrett et al. 2007; Flynn et al. 2002; Matsusaki et al. 2006; Ocaña et al. 1999; Pilasombut et al. 2006). In this study, the first reaction of the PCR walking technique was performed using the primers SalAα-f1 and SalAα-r1 resulting in one ORF named *kld*1 which exhibits 100 % identity to the gene encoding for the uncharacterized bacteriocin α peptide of *Lb. salivarius* GJ-24 (Accession No. EGM49654.1). However, it displayed 94 % identity to the gene encoding Abp118α of *Lb. salivarius* (Accession No. AAM61778.1). The *kld*1 sequence of *Lb. salivarius* KL-D4 was submitted into GenBank of the NCBI database with Accession No. KT307081 on July 16th, 2015. Its molecular weight of 4.202 and isoelectric focusing point of 9.02 (by a peptide property calculator program) were different to other salivaricins α of 4.0 and 8.58, respectively (Barrett et al. 2007; Cho et al. 2011; Flynn et al. 2002; Matsusaki et al. 2006; Ocaña et al. 1999; Pilasombut et al. 2006).

Further walking was done using the primer D4-f1 and Oligo105 designed from *kld*1 and the degenerate primer based on histidine kinase of class II bacteriocin (Eijsink et al. 2002; Nissen-Meyer et al. 2010; Oppegrad et al. 2007), respectively, resulting in a 1.8 kb PCR fragment which contained three ORFs named *kld*2, *kld*3 and *kld*4. By blast analysis, the *kld*2, *kld*3 and *kld*4 showed identity of 100, 100 and 99 % to the gene encoding uncharacterized bacteriocin β peptide (Accession No. EGM49653.1), uncharacterized hypothetical protein (Accession No. EGM49652.1) and Abk transduction sensory histidine protein kinase (Accession No. EGM49651.1) of *Lb. salivarius* GJ-24 of *Lb. salivarius* GJ-24, respectively. It seemed that the gene cluster of the strain KL-D4 was similar to the one of GJ-24. However, those *kld*2, *kld*3 and *kld*4 displayed different identities to Abp118β (Accession No. AAM61779.1), the salivaricin induction peptide (Accession No. ABQ84445.1) and the histidine protein kinase (Accession No. AAM61782.1) for 93, 59 and 62 % of other *Lb. salivarius* strains, respectively.

To investigate other genes, inverse PCR was performed. Chromosomal DNA of *Lb. salivarius* KL-D4 was randomly digested by 9 restriction enzymes. Only *Sac*I digested chromosomal DNA was successfully amplified by the primers D4-f1 and D4-r1 resulting in 2.3 kb containing an open reading frame of 294 bp named *kld*5 and the other pMD-20T vector sequences. The *kld*5 exhibited 99 % similarity to the gene coding unclarified bacteriocin family from *Lb. salivarius* GJ-24 (Accession No. EGM49655.1) but not to other salivaricin producing strains.

**Fig. 2:**
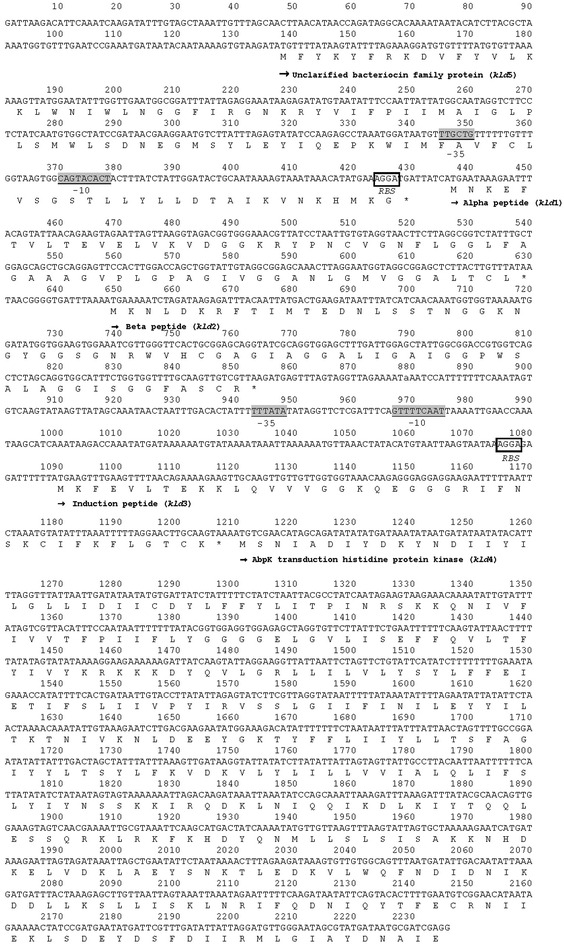
Nucleotide sequence and deduced protein in the *kld* locus. The putative promoter sequences are *underlined*; the ribosomal binding sites (RBS) are *boxed* and *underlined*. Nucleotide sequences alignment was performed using the GENETYX-MAC software (GENETYX^®^, Japan) on September 28th, 2012. Bacterial promoter prediction was performed using BROM on the Softberry (online program) on August 4th, 2015

According to the results above, it could be summarized that *Lb. salivarius* KL-D4 has two structural bacteriocin genes similar to Abp118 α and β related to the salivaricin family and another two genes to regulation of class-II bacteriocin. Adjacency to the salivaricin gene cluster and confirmation by the primer D4-f2 and the antisense primer D4-r2 are shown in Fig. 2. Two promoter and ribosomal binding sites (RBS) located on the upstream region of *kld*1 and *kld*3 were found. An open reading frame (ORF) analysis performed using an ORF Finder program confirmed that two structural genes of *kld*1 and *kld*2 were located on different ORF frames of +1 and +3, respectively. It seemed that *kld*2 did not have its own promoter and RBS for gene expression. It was, therefore, proposed that bacteriocin was expressed by only *kld*1.

### Purification of bacteriocin produced by *Lb. salivarius* KL-D4

**Fig. 3:**
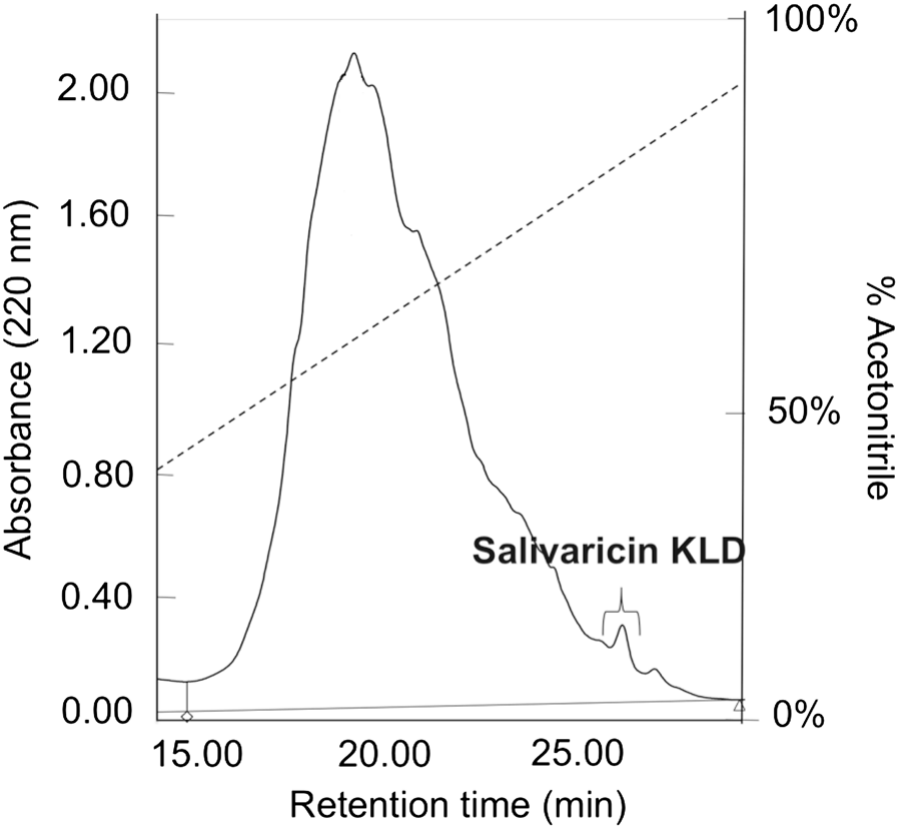
Reverse-phase chromatogram of the salivaricin KLD. The target fraction (a salivaricin KLD fraction) was earned by eluting with 74 % of solvent B. *Dash line* is a gradient condition of solvent B (acetonitrile, %v/v)

**Fig. 4:**
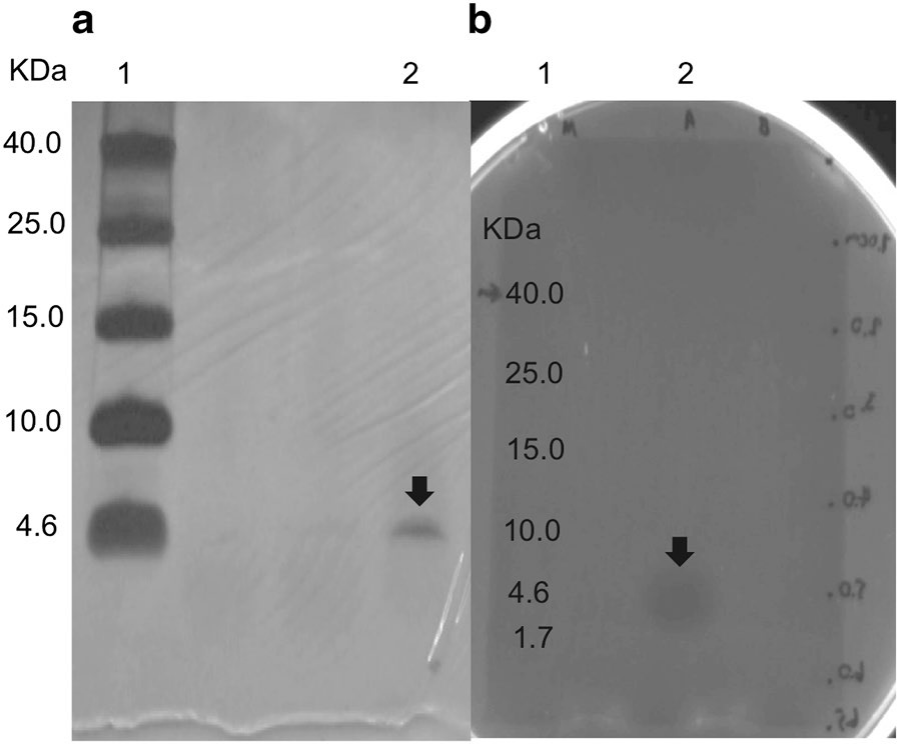
Characterization of purified salivaricin KLD. Silver stained Tris–Tricine SDS-PAGE containing 15.0 % polyacrylamide gel (**a**), Protein with antibacterial activity visualized by overlaying soft MRS agar containing cells of *Lb. sakei* subsp. *sakei* JCM 1157^T^ (**b**); *lane 1* Spectra Multicolor Low Range Protein Ladder (Thermo Scientific Pierce, USA); *lane 2*: purified salivaricin KLD. The *arrows* indicate a salivaricin KLD fraction and its activity

Purification of bacteriocin was achieved by three purification steps as shown in Table 6. Partial purification by 20 % ammonium sulfate saturation and cation exchange chromatography provided only 1 % yield. The pure bacteriocin was eluted from the reverse phase HPLC with 74 % solvent B (Fig. 3) to obtain purification and yields of 40.68 folds and 0.01 %, respectively. By Tris–Tricine SDS-PAGE analysis, only a single protein band of about 4.3 kDa was obtained as shown in Fig. 4, namely salivaricin KLD.

**Table 6:** Purification table of salivaricin KLD

Purification stage	Vol. (ml)	Activity (AU/ml)	Protein concentration (mg/ml)	Total protein (mg)	Total activity (AU)	Specific activity (AU/mg)	Purification (folds)	Yield (%)
Cell free supernatant	1000	25,600	18.600	18,600.000	2.56 × 10^7^	1.38 × 10^3^	1	100
(NH_4_)_2_SO_4_ precipitate	20	102,400	2.150	43.000	2.05 × 10^6^	4.77 × 10^4^	34.57	8.01
Cation exchange	20	12,800	0.224	4.480	2.56 × 10^5^	5.71 × 10^4^	41.38	1.00
RP-HPLC	1	3200	0.057	0.057	3.20 × 10^3^	5.61 × 10^4^	40.68	0.01

All % yield values are expressed in terms of activity units in the cell free supernatant taken as 100 % while purification values are in the terms of specific activities in the cell free supernatant taken as 1

### Effect of salivaricin KLD on the growth of bacterial contaminants in creamy filling

The partially purified salivaricin KLD using ammonium sulfate precipitation and freeze-drying that exhibited inhibitory activity against bacterial contaminants from the production process of éclair was studied. Six target strains isolated from the production process of E’clair product were used to perform the experiments and the results are summarized in Table 7. The growth inhibitory efficiency (GIE) of 1, 5 and 10 % of KLD compared to the efficiency of two commercial bacteriocins, 1 % H3 and 2 % glycine, were tested at 0 and 3 days as shown in Table 7. Various concentrations of KLD showed high GIE values of 22–39 and 52–100 % against *E. faecalis* KUB-E5 at 0 and 72 h, respectively, while both the commercial ones displayed only 0.39–1.8 %. The activity of KLD against *E. faecalis* KUB-E7 was lower than against *E. faecalis* KUB-E5; however, its activity against *E. faecalis* KUB-E7 was still higher than for the other commercial compounds.

**Table 7:** Growth inhibitory efficiency of antimicrobial substances against bacterial contaminants in sterilized creamy filling containing about 5 log CFU/g of each target strain and various food preservatives of PP-KLD, H-3 and glycine

Target strain	Treatment % (w/w)^a^	Growth inhibitory efficiency (%)^b^	
		0 h	72 h
*Enterococcus fecalis* KUB-E5	1 %PP-KLD	22.76 ± 0.82b	52.27 ± 2.09b
	5 %PP-KLD	39.30 ± 3.08c	100.00 ± 0.00c
	10 %PP-KLD	26.46 ± 0.40b	100.00 ± 0.00c
	1 %H-3	0.39 ± 0.16a	1.75 ± 0.27a
	2 %Glycine	1.27 ± 0.32a	1.87 ± 0.45a
*Enterococcus fecalis* KUB-E7	1 %PP-KLD	15.40 ± 3.14b	11.51 ± 5.34b
	5 %PP-KLD	17.83 ± 2.43b	30.69 ± 0.28c
	10 %PP-KLD	19.96 ± 0.94b	60.12 ± 0.4d
	1 %PP-H-3	1.88 ± 0.08a	0.90 ± 0.13a
	2 %PP-Glycine	3.61 ± 0.39a	3.89 ± 0.15a
*Staphylococcus epidermidis* KUB-E6	1 %KLD	1.83 ± 1.02b	0.00 ± 0.00a
	5 %KLD	0.46 ± 2.36a	0.00 ± 0.00a
	10 %KLD	1.80 ± 2.55b	0.00 ± 0.00a
	1 %H-3	3.24 ± 0.38c	24.58 ± 1.11b
	2 %Glycine	0.00 ± 0.00a	22.37 ± 1.14b
*Staphylococcus hominis* KUB-E8	1 %PP-KLD	1.96 ± 1.95a	22.91 ± 0.23a
	5 %PP-KLD	3.85 ± 2.66a	28.10 ± 1.38c
	10 %PP-KLD	3.37 ± 0.17a	25.80 ± 1.85c
	1 %H-3	2.58 ± 0.18a	23.05 ± 0.28ab
	2 %Glycine	10.54 ± 0.27b	25.69 ± 0.28bc
*Bacillus cereus* JCM2152	1 %PP-KLD	2.02 ± 1.43a	4.47 ± 2.43a
	5 %PP-KLD	6.18 ± 0.41b	50.39 ± 1.58b
	10 %PP-KLD	10.14 ± 0.29c	66.94 ± 0.88c
	1 %H-3	3.28 ± 0.19a	44.29 ± 0.29b
	2 %Glycine	8.85 ± 0.22c	6.97 ± 0.17a
*Pseudomonas stutzeri* KUB-E1	1 %PP-KLD	3.92 ± 5.93a	100.00 ± 0.00c
	5 %PP-KLD	100.00 ± 0.00c	100.00 ± 0.00c
	10 %PP-KLD	100.00 ± 0.00c	100.00 ± 0.00c
	1 %H-3	11.35 ± 1.15b	12.53 ± 0.58a
	2 %Glycine	14.96 ± 0.31b	64.07 ± 2.34b
*Stenotrophomonas* sp. KUB-E2	1 %PP-KLD	0.27 ± 0.16a	5.25 ± 2.56a
	5 %PP-KLD	0.00 ± 0.00a	100.00 ± 0.00d
	10 %PP-KLD	1.50 ± 0.40b	100.00 ± 0.00d
	1 %H-3	5.82 ± 0.05c	9.04 ± 0.36b
	2 %Glycine	6.24 ± 0.54c	44.84 ± 1.44c

All growth efficiency obtained are a mean of two replications ± SD (SD); a–d, means in the same column of each target strain with different lower case letters are significantly different (*P* < 0.05)

^a^Five treatments of antimicrobial substance PP-KLD at the concentrations of 1, 5 and 10 %, 1 % H3 and 2 % glycine tested

^b^Growth inhibitory efficiency defined as percentage of survival cells after antimicrobial substance treatment compared to the control mentioned elsewhere

Two species of *Staphylococcus* (*S. epidermidis* KUB-E6 and *S. hominis* KUB-E8) were investigated. The 5 and 10 % concentrations of KLD and the 2 % glycine treatment exhibited significantly higher GIE than the other treatments against the growth of only strain KUB-E8, while the KLD showed no activity to the strain KUB-E6 at 72 h.

*Bacillus cereus* is a frequent contaminant in bakery products and causes rope spoilage. It was found that *B. cereus* JCM 2152 had significantly (*P* < 0.05) the highest sensitivity to 10 % KLD resulting in GIE values of 10.14 ± 0.29 and 66.94 ± 0.88 % at 0 and 72 h, respectively, while the activities of 5 % KLD and 1 % H3 showed no significant difference of GIE at 72 h.

Considering the two Gram-negative bacterial strains of *Pseudomonas stutzeri* KUB-E1 and *Stenotrophomonas sp.* KUB-E2, *P. stutzeri* KUB-E1 was more sensitive to 5 and 10 % of KLD at both 0 and 72 h while 1 % KLD exhibited 100 % GIE at 72 h. Both 5 and 10 % KLD also showed 100 % GIE against *Stenotrophomonas sp.* KUB-E2 at 72 h. Both commercial AMS products (H3 and glycine) had lower GIE levels against both Gram-negative bacteria compared to the KLD.

## Discussion

A salivaricin KLD produced by *Lb. salivarius* KL-D4 showed high resistance to heat, high stability over a wide pH range of 3.0–10.0, and a wide antibacterial spectrum against Gram-positive and Gram-negative bacteria. In addition, it should be noted that the salivaricin KLD is stable at high pH of 8–10, while nisin, a well-known bacteriocin usually used in the bakery products rapidly loses its activity at a pH of more than 4 (Rollema et al. 1995). The salivaricin KLD also effectively inhibited spore-forming *B. cereus*, which often contaminates bakery products, such as custards and cream cakes (Siriken et al. 2009). Therefore, these characteristics are promising for applications of the salivaricin KLD in the creamy production process, as well as in the other bakery lines, which involve alkaline or non acid products.

The salivaricin KLD production was enhanced by culture broth supplemented with 0.5 % (w/v) CaCO_3_ under gentle shaking. The maximum production was observed in the early stationary phase and was maintained until later stages. Its production was growth-associated and displayed as primary metabolite kinetic which was similar to almost all bacteriocins produced by lactic acid bacteria (Cheigh et al. 2002; Zamfir et al. 2000). Neutralization of lactic acid in the culture by CaCO_3_ described by Matsusaki et al. (1996) and increasing the CaCO_3_ diffusion by gentle shaking could enhance its growth and bacteriocin production as supported by Yamamoto et al. (2003). However, the antibacterial activities observed subsequently decreased in the later stages perhaps due to extracellular proteases produced and acidity during fermentation (Parente and Ricciardi 1999). Bioactivity is usually limited to a narrow time interval of maximum fermentation (about 10–12 h), due to low pH conditions from the accumulation of lactic acid contents or an exhausted energy source. Then, cell growth and the bacteriocin production will gradually stop. Therefore, both the pH value and lactic acid production influenced both the cell growth and the bacteriocin-production kinetics, which must be controlled. The genes responsible for the salivaricin KLD production were located on the 2.3 kb DNA fragment. The cluster of putative genes required for the salivaricin KLD production was similar to other class-IIb bacteriocin gene clusters (Nes et al. 1996). The gene *kld*1 and *kld*2 encoding for pre-peptides salivaricin α and β from *Lb. salivarius* KL-D4 showed high similarity to the ones from *Lb. salivarius* GJ-24 isolated from the feces of healthy adults without the characteristics of those peptides reported (Cho et al. 2011). To date, several salivaricins with high structural similarity produced by *Lb. salivarius* have been isolated from the intestinal tracts of humans and animals, such as salivaricin ABP-118 produced by the strain UCC118 isolated from human intestines (Flynn et al. 2002), salivaricin CRL1328 produced by the strain CRL1328 isolated from healthy human vaginal samples (Ocaña et al. 1999), salivaricin P produced by the strain DPC6005 isolated from a porcine intestine (Barrett et al. 2007), salivaricin FK22 and salivacin K21 produced by the strain K7 (Pilasombut et al. 2006) and KUB-AC21 isolated from chicken intestines (Matsusaki et al. 2006). Modification of their amino acid sequences occurred depending on the environment of each source and resulted in only 94 % identity to salivaricin KLD which led to different inhibitoty activity against the growth of the Gram-negative bacteria, *Pseudomonas* and *Stenotrophomonas*. To date, no salivaricin from other producer strains have been reported regarding their activity against Gram-negative bacteria.

The genes encoding the KLD3 and KLD4 found were located on the DNA fragment and were involved in bacteriocin production called a three-component regulatory system, that includes peptide pheromone (induction peptide, IF) and histidine protein kinase (HPK) (Diep et al. 2003; Eijsink et al. 2002; Kleerebezem and Quadri 2001). However, *kld*3 showed only low identity to the peptide pheromone reported, while it induced higher bacteriocin production of up to 25,600 AU/ml compared to the other *Lb. salivarius* strain reported. This supported a strong induction peptide coded by *kld*3. However, other genes related with immunity were not observed by this study. Salivaricin KLD was previously tested for its inhibitory activity against its own producer strain resulting in no inhibition. Therefore, the gene encoding IM of the KL-D4 strain may exist but be located elsewhere on chromosomal DNA, which is different to salivaricin APB-118 (Flynn et al. 2002), salivaricin P (Barrett et al. 2007), salivaricin CRL1328 (Vera Pingitore et al. 2009), and salivacin K21 (Matsusaki et al. 2006) which are located near the structural salivaricin peptide gene.

Only salivaricin KLD similar to α peptide was expressed and produced by *Lb. salivarius* KL-D4. This result was similar to Messaoudi et al. (2013) and Flynn et al. (2002) who reported the detection of α peptide in their purification process, while β peptide could be produced by *Lb. salivarius* K7 (Pilasombut et al. 2006). However, both α and β peptides were produced by *Lb. salivarius* DPC6005 and successfully purified by RP-HPLC (Barrett et al. 2007).

The salivaricin KLD showed high stability within the pH range 5.5–7.0. These properties would make it useful as a biopreservative in low acid food such as bakery products classified as non-acidic (Smith et al. 2004). In a tropical country like Thailand, the contaminant cell concentration can be as high as up to 5 log CFU/g in cream-filled products which differs from the European zone where lower numbers of 1–4 log CFU/g have been reported (Leitenberger and Rocken 1998; Siriken et al. 2009). PP-KLD showed remarkable effective inhibition to contaminant strains of *B. cereus*, *E. fecalis*, *P. stutzeri*, as well as *Stenotrophomonas* sp. Furthermore, the effective inhibition was superior to the commercial bacteriocins of H-3 and glycine. However, both PP-KLD and the commercial bacteriocins were not active against *Staphylococcus* species. This might have been negatively influenced by the high incubation temperatures promoting faster growth of *Staphylococcus* (Viedma et al. 2009), which corresponded with the antimicrobial substance PP-174 produced by *Lb. plantarum* KJ-174 showing low inhibitory efficiency in cream filling against aerobic mesophilic bacteria, when the incubation temperature was increased from 15 to 37 °C (Wongsuttichote and Nitisinprasert 2009). To solve this problem, combined preservation called hurdle technology (Cleveland et al. 2001) will be needed to achieve successful preservation in future work.

## Conclusion

Salivaricin KLD shares functional properties of the bacteriocin α peptide that is heat tolerant, stable at a wide pH range, and exhibited widely antibacterial spectrum. Partial purification by 20 % ammonium sulfate precipitation of the product named PP-KLD was tested on the artificial contamination of creamy filling. The 5 % PP-KLD (w/w) exhibited effective growth inhibitory efficiency against *Pseudomonas* sp., *P. stutzeri*, *Stenotrophomonas* sp. and some strain of *E. faecalis* but showed low activities against *B. cereus*, *Staphylococcus epidermidis* and *Staphylococcus hominis*. To complete inhibitory activity against microbial contaminants, cooperation with other hurdle technology may be considered in the future.
